# Quantifying Physical Thrombus Characteristics on Cardiovascular Biomaterials Using MicroCT

**DOI:** 10.3390/mps3020029

**Published:** 2020-04-13

**Authors:** Avi Gupta, Claire M. Johnston, Monica T. Hinds, Deirdre E. J. Anderson

**Affiliations:** Department of Biomedical Engineering, Oregon Health & Science University, Portland, OR 97239, USAhindsm@ohsu.edu (M.T.H.)

**Keywords:** hemocompatibility, thrombus, microCT, biomaterials, vascular graft, platelets, fibrin, arteriovenous shunt

## Abstract

Hemocompatibility is a critical consideration when designing cardiovascular devices. Methods of assessing hemocompatibility range from in vitro protein adsorption and static platelet attachment to in vivo implantation. A standard preclinical assessment of biomaterial hemocompatibility is ex vivo quantification of thrombosis in a chronic arteriovenous shunt. This technique utilizes flowing blood and quantifies platelet accumulation and fibrin deposition. However, the physical parameters of the thrombus have remained unknown. This study presents the development of a novel method to quantify the 3D physical properties of the thrombus on different biomaterials: expanded polytetrafluoroethylene and a preclinical hydrogel, poly(vinyl alcohol). Tubes of 4–5 mm inner diameter were exposed to non-anticoagulated blood flow for 1 hour and fixed. Due to differences in biomaterial water absorption properties, unique methods, requiring either the thrombus or the lumen to be radiopaque, were developed to quantify average thrombus volume within a graft. The samples were imaged using X-ray microcomputed tomography (microCT). The methodologies were strongly and significantly correlated to caliper-measured graft dimensions (*R*^2^ = 0.994, *p* < 0.0001). The physical characteristics of the thrombi were well correlated to platelet and fibrin deposition. MicroCT scanning and advanced image analyses were successfully applied to quantitatively measure 3D physical parameters of thrombi on cardiovascular biomaterials under flow.

## 1. Introduction

Interest in cardiovascular biomaterials is primarily driven by the growing prevalence and mortality of cardiovascular disease. Vascular graft technologies, including arterio-venous grafts in hemodialysis access for patients with kidney failure, peripheral artery disease revascularization, and coronary artery bypass grafting (CABG), have a significant clinical impact. CABG procedures neared 400,000 in 2014 [1]. Although saphenous veins are the most common graft material and are more clinically effective than synthetic grafting with currently available materials, many bypass patients do not have suitable autologous veins for grafting [2,3]. Current synthetic grafts in clinical use include expanded polytetrafluoroethylene (ePTFE) and polyethylene terephthalate (Dacron). These materials are desirable for their bioinert and hydrophobic properties. However, they exhibit poor long-term patency rates. Despite the poor long-term results for both ePTFE and Dacron grafting, no new grafting solutions have been approved by the United States Food and Drug Administration pre-market approval process in over 10 years, in large part due to the lack of hemocompatibility of the materials. 

An essential criteria for any vascular graft is hemocompatibility to prevent thrombosis, a primary cause of graft failure [4]. As reviewed previously, the process of biomaterial-induced thrombosis is mediated by the binding of platelets to the rapidly adsorbed proteins on the biomaterial or tissue-engineered surface [5]. Thrombosis has been particularly well-studied in vitro. For example, many groups have studied biomaterial hemocompatibility using isolated coagulation factors or purified platelets [6,7]. More complex systems include using plasma alone or platelet-rich plasma (PRP) in vitro to determine platelet–surface interactions. Although these studies provide important mechanistic insights, a more complete system for studying thrombosis requires whole blood to understand the roles and interactions of the coagulation factors and platelets. Whole blood testing with the ability to control anticoagulant intervention is reflective of the physiological conditions of clinical graft implantations. Therefore, whole blood testing of biomaterials provides a more accurate assessment of the hemocompatibility and of the potential performance in clinical applications [8]. 

Our group has used a whole blood, ex vivo shunt model for many decades to evaluate the hemocompatibility of cardiovascular biomaterials [8,9,10,11,12,13]. The ability to control flow rate and local or systemic drugs makes the ex vivo whole blood shunt model very powerful for potential clinical translation. These studies have generated dynamic, quantitative platelet and endpoint fibrin data for numerous biomaterials and experimental drugs. Yet, despite an extensive body of work investigating hemocompatibility of cardiovascular biomaterials and tissue-engineered surfaces, little is understood about the physical properties of the thrombi formed under these ex vivo conditions. The physical thrombus properties are critical to the patency of a vascular graft. However, two-dimensional physical or microscopic inspection, with or without a highly subjective score, is frequently the extent of assessment of the physical parameters of a three-dimensional thrombus [14,15]. In order to determine the potential clinical efficacy of novel biomaterials, an improved method for the assessment of graft thrombogenicity under ex vivo conditions is required. The development of such a method will better predict patency and therefore device success or failure.

In this study, microcomputed tomography (microCT) imaging was used to analyze thrombus formation in small-diameter vascular grafts formed under flowing whole blood, primarily without antiplatelet therapies, in ex vivo shunt studies. Multiple biomaterials were used to induce a range of sizes of the thrombus. Specifically, ePTFE served as a clinical control; collagen-coated ePTFE acted as a positive, thrombogenic control; and poly(vinyl alcohol) (PVA) served as a preclinical, non-thrombogenic, hydrogel material. Several surface modifications of the PVA, which were previously evaluated for their hemocompatibility and endothelialization potential [16,17,18,19], were used in this work to assess and validate the measurement of the physical properties of the thrombus. Advanced image software analysis was then used to quantify the luminal volume (the inverse of thrombus volume) and the intra-thrombus variability. We hypothesized that the quantification of platelet and fibrin attachment would inversely correlate to the luminal volume derived using image analysis. That is, an increase in platelets or fibrin would correspond to an increase in thrombus volume and thus decrease in the volume of the lumen. This study quantified the extent of this correlation and developed a novel methodology for providing key quantitative data on the hemocompatibility of cardiovascular biomaterials. These techniques have broader applications to any number of biomaterials or tissue-engineered vascular grafts and have the potential to significantly impact the development of clinical grafts or other cardiovascular devices. 

## 2. Materials and Methods

Overall, to demonstrate this method, multiple biomaterials were used to induce a range of thrombus sizes. The water absorption of the materials necessitated the use of multiple approaches to render the lumen or thrombus radiopaque and quantify the physical properties of the thrombus.

### 2.1. Device Manufacturing and Preparation

Collagen-coated ePTFE samples served as a positive thrombosis control and were prepared as described previously [20]. Briefly, the ends of 2 cm segments of ePTFE were attached to silicone tubing (Technical Products, Inc, Oxford, GA, USA) with silicone glue. Additional glue was added to the outer surface of the materials to reinforce the ends. The grafts were covered in heat shrink tubing and the lumen was coated with equine type I collagen (1 mg/mL; Nycomed Arzeneimittel, Munich, Germany) [21]. Unmodified ePTFE vascular grafts (W.L. Gore) were used as clinical controls (7 cm in length), and were also fabricated in accordance with previous descriptions [11,21]. Samples of 3 or 4 cm in length were cut out of the graft after the ex vivo shunt whole blood test to avoid edge effects in the analysis of the thrombus.

Grafts of our preclinical hydrogel (PVA) were manufactured as described previously [11]. A solution of 10% aqueous PVA (Sigma-Aldrich, 85–124 kDa, 87%–89% hydrolyzed) was combined with 15% (*w*/*v*) sodium trimetaphosphate (STMP) (Sigma Aldrich) cross-linker and 30% (*w*/*v*) NaOH. Rods of 4 mm were dip cast to form 4 to 5 mm tubes after delamination. The luminal surfaces were then modified using a variety of techniques, including biochemical and topographical modifications, and assessed for thrombogenesis and hemocompatibility in the ex vivo shunt whole blood test. Specific modifications included integration of the peptide sequence Gly-Phe-Pro-Gly-Glu-Arg (GFPGER), plasma modification, addition of gelatin with a carbonyldiimidazole (CDI) covalent linker, and sterilization, and their preparation methods are described in greater detail in previous publications [16,17,18,19]. Samples, which were modified by the integration of GFPGER, a peptide subsequence of type I collagen, had GFPGER mixed with the STMP in the PVA manufacturing process [17]. The luminal surfaces of plasma modification samples were subjected to NH_3_ plasma modification using a variation of the radio frequency glow discharge treatment in the presence of NH_3_, as described previously [16,22]. The PVA for the gelatin-modified samples were activated using CDI with the goal of conjugating a gelatin protein, as described previously [18]. Additionally, unmodified PVA samples were subjected to terminal sterilization using ethylene oxide (EtO) and gamma radiation after cross-linking and before implantation, as described previously [19]. Following manufacturing and surface modifications, PVA grafts were assembled for testing by attaching each graft to silicone tubing (Technical Products, Inc.) using heat shrink (Small Parts, Inc.) tubing connectors. Parafilm (Bemis) was used to envelop the connectors to strengthen the junction, as described previously [11]. Samples of 4 cm in length were cut out of the center of the graft after the ex vivo shunt whole blood test to avoid edge effects in the analysis of the thrombus.

### 2.2. Whole Blood Testing

**Figure 1:**
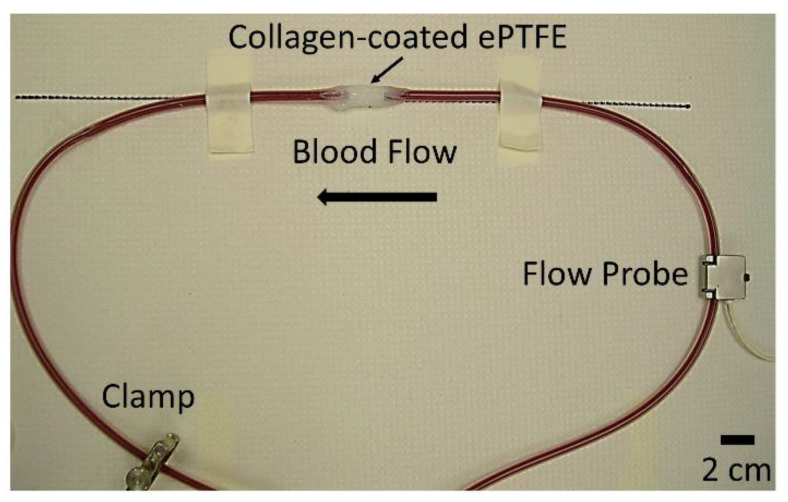
Apparatus for ex vivo arteriovenous shunt loop whole blood testing. In this case, a 2 cm length, 4 mm inner diameter collagen-coated expanded polytetrafluoroethylene (ePTFE) sample in the testing position is shown. Scale bar = 2 cm.

Overall, 110 PVA samples, 26 ePTFE samples, and 20 collagen-coated ePTFE samples were tested and analyzed for this study. All grafts with extended silicone tubing were placed in an ex vivo, baboon, femoral, arteriovenous shunt loop (Figure 1). Samples were tested in five different animals. For a small subset of samples [17], acetylsalicylic acid (ASA, Bayer Healthcare) or ASA and clopidogrel (Torrent Pharmaceuticals) was given before experimentation to reduce thrombus formation. Specifically, there were six PVA grafts, three ePTFE grafts, and one collagen-coated ePTFE graft treated with ASA; six PVA grafts, three ePTFE grafts, and one collagen-coated ePTFE grafts were treated with ASA and clopidogrel. ASA was dosed orally at 10 mg/kg at least 4 h before the start of each experiment, and clopidogrel was dosed twice per day orally at 2 mg/kg throughout the length of experimentation. The thrombogenicity of the surface was tested with whole blood containing radiolabeled platelets and fibrin. Platelet and fibrin accumulation were quantified as described previously [8]. Briefly, indium-111-labeled platelets and iodine-125-labeled fibrin were introduced into the baboons. Platelet deposition was measured throughout the 1-hour study using a gamma camera. Fibrin incorporation was quantified following the total decay (>10 half-lives) of indium-111 [11]. Juvenile male baboons were cared for according to strict internal and external regulations, which were described in detail previously [8].

Upon completion of the ex vivo study (whole blood testing), grafts were fixed for subsequent microCT scanning. For ePTFE samples, a larger diameter rigid tubing was added to the exterior of the device to reduce shrinking during fixation. All samples were rinsed with phosphate-buffered saline (PBS) with Ca^2+^ and Mg^2+^, and 3.7% paraformaldehyde (PFA) was added. The materials were refrigerated for 24–72 hours to allow for complete fixation. The samples were then rinsed with PBS to remove the PFA. The ePTFE samples were stored in PBS until the day before microCT scanning.

### 2.3. MicroCT Preparation and Imaging

Following fixation, the samples were processed to make either their lumen (PVA) or thrombus (ePTFE, collagen ePTFE) radiopaque for microCT imaging. Each method was evaluated for each of the materials before selecting the methods presented here. Due to the differing material properties of the hydrogel (PVA) and the ePTFE, different methods were used to achieve radiopacity. The lumens of the PVA grafts were filled with Microfil^®^ (Flow Tech, Inc.), which was mixed according to the manufacturer’s instructions. Prior to Microfil^®^ addition, a syringe was used to remove any excess liquid in the grafts. The Microfil^®^ was slowly pipetted into the lumen of the graft with special care taken to avoid air bubbles in the lumen. The Microfil^®^ was cured for 2 hours at room temperature. After curing, the Parafilm and heat-shrink tubing connectors were cut away from the grafts, which were trimmed to their middle 4 cm. PVA samples were stored in PBS until microCT scanning. The thrombus within the ePTFE and collagen-coated ePTFE grafts were rendered radiopaque by soaking in Lugol’s solution (Sigma) for approximately 16 hours prior to microCT imaging. 

The grafts were scanned using microCT (Perkins-Elmer, Caliper QuantumFX) with a consistent orientation of the proximal and distal graft ends. Immediately prior to scanning, the glue coating was removed from the collagen-coated ePTFE samples. The samples were centered in the scanner’s field of view using the “Live” mode and built-in stage adjustment. The 2 min (“Fine”) scan was performed using a current of 180 µA, voltage of 90 kV, and 60 mm field of view. With this instrumentation, operated at 30 frames per second (2 × 2 binning, 512 × 512 matrix), there were 3672 acquired projections, which, after four-projection averaging, resulted in 918 projections in the reconstructed image. The exposure time per projection was 0.03327 s. The resultant voxel (117 × 117 × 117µm) images were used for analysis. The sample images ranged from 165–369 slices, depending on the length of the graft. After imaging, sample dimensions (length, outer diameter, inner diameter, and wall thickness) were measured using digital calipers.

### 2.4. Image Analysis and Processing

To determine the physical characteristics of the thrombi on the basis of the microCT images, the Amira software package (FEI, version 5.2.2) was used by a trained operator, blinded to the specific sample treatment. The software is capable of generating three-dimensional models of the radiopaque regions of each graft, as well as calculating volume and length. Voxel images produced by the microCT scanner were imported into Amira with defined dimensions. Specifically, voxel size was calculated by dividing the field of view (FOV) in millimeters (60 mm in this case) by the voxel quantity (512). After the image was loaded, a label file was created in Amira for each image. Label files allowed for selection of specific regions of interest (ROIs) in each cross-sectional luminal slice of the image. These ROIs were combined into a final volume-rendered surface. A summary of the methods used for the various material types is provided in Table 1.

**Figure 2:**
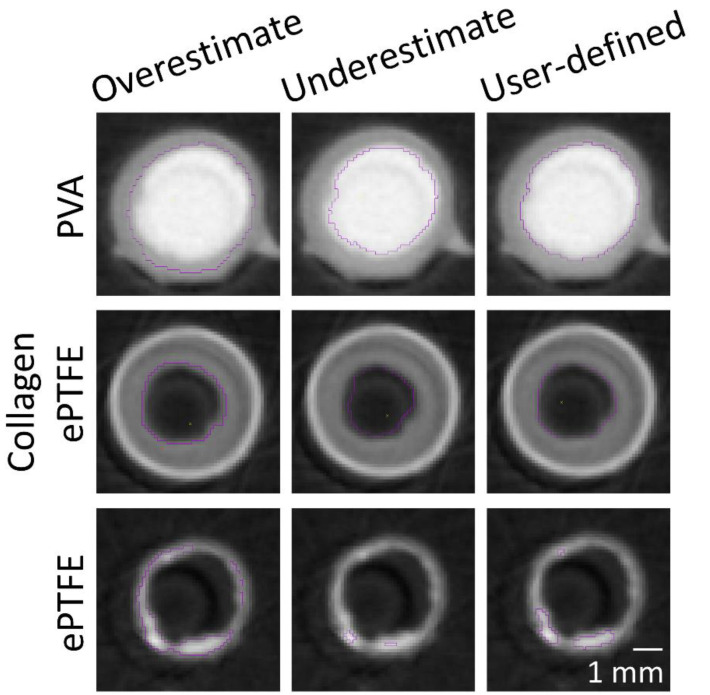
Cross-sectional images of 4mm inner diameter biomaterial graft samples with the luminal area highlighted by the user-selected masking range (purple line). These calibrations were used as a guideline for users to select regions of interest (ROIs) in Amira, which were combined to generate the final surface. Scale bar = 1 mm for all images.

The masking wand within Amira was used to select the ROIs to include all pixels contiguous to the blinded user’s mouse click with intensity within a user-specified range. As the selection of a masking intensity range greatly impacted the final surface volume, standard calibrations were developed to ensure consistency across users and analyses. All masking ranges for PVA samples were selected to include the interior of the bright radiopaque central lumen, as well as one layer of medium gray border pixels on the outside edge (Figure 2). A similar standard was applied for collagen-coated ePTFE samples, although the interior lumen of these samples was black. For ePTFE samples with or without collagen, the thrombi were rendered radiopaque by their absorption of the Lugol’s solution during the soaking process prior to microCT scanning. These thrombi were then selected as the region of interest in the Amira software by selecting a masking range that included only pixels with the higher intensity. 

**Figure 3:**
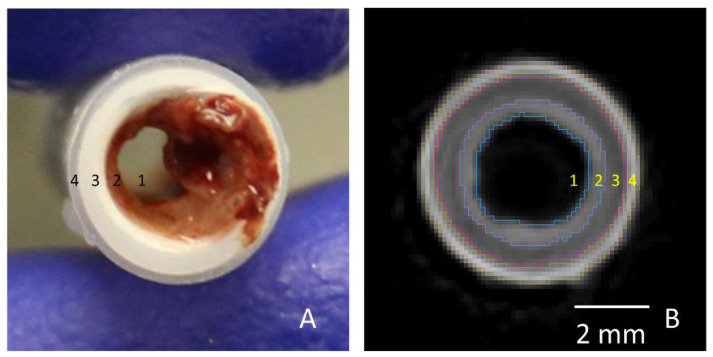
Cross-sectional images of two different collagen-coated ePTFE (ID = 4 mm) samples including all four labeled materials: the lumen (1), the thrombus (2), collagen-coated ePTFE biomaterial (3), and heat shrink tubing (4). Image taken after fixation is shown on the left (**A**) and a cross-section from the materials segmentation in Amira is shown on the right (**B**). Scale bar = 2 mm.

For PVA samples, the ROI contained the luminal area of the graft in each cross-section. For ePTFE samples, ROIs were selected to contain the thrombi in each cross-section. For the collagen-coated ePTFE samples, multiple ROIs (luminal and thrombus areas) were selected. The Amira imaging software allows the user to select multiple “materials” for a single sample. First, the luminal area for each cross-section was selected similar to the PVA samples. Next, the masking regions were adjusted to select the outermost heat shrink tubing as a new material. Then, the ePTFE was selected. Finally, the remaining material, which was the thrombus, was selected (Figure 3). 

The luminal volumes for PVA and collagen-coated ePTFE samples were directly quantified using selective pixel intensity masking in the Amira software. For ePTFE samples, luminal volume was calculated by subtracting the thrombus volume quantified in the Amira software from a volume calculated using a cylindrical approximation on the basis of caliper measurements. For all samples, the luminal volume was then used to calculate the average luminal area through length normalization based on the Amira-measured length; thus, results are presented as average luminal volume per length, or average cross-sectional area.

After ROIs were selected for all cross-sections, the length of the resultant surface was obtained using Amira’s slice-numbering system. The cross-sectional ROIs were then combined using Amira’s “SurfaceGen” feature to yield a three-dimensional representation of either the lumen (PVA, collagen ePTFE) or the thrombus (ePTFE, collagen ePTFE) of the graft. The areas of these surfaces within a slice were then measured using Amira’s “SurfaceArea” tool. To gain a more detailed understanding of the thrombus geometry over the entire graft surface, samples were examined on the basis of each cross-sectional slice datum, as imaged by the microCT and generated by the Amira software. The the cross-sectional thrombus and luminal areas over the entire length were exported into a table using Amira’s “AreaPerSlice” tool. 

### 2.5. Implant Testing

These methods were applied in a single implant study in a single animal. An aortoiliac bypass study was performed as described previously, according to the ‘‘Guide for the Care and Use of Laboratory Animals’’ prepared by the Committee on Care and Use of Laboratory Animals of the Institute of Laboratory Animal Resources, National Research Council, with approval by the Institutional Animal Care and Use Committee [8,23]. In brief, PVA and ePTFE grafts were implanted bilaterally from the aorta of a male, juvenile baboon to the iliac artery using end-to-side anastomoses. After 28 days, samples were explanted, fixed for several days, and soaked in Lugol’s solution overnight. The sample was imaged with the MicroCT and imported into Amira as described above. Because all the tissue was rendered radiopaque by the Lugol’s, the open areas were selected to illustrate the lumens of the vessels and graft. Multiple “materials” were selected to segment arterial or venous flow. 

### 2.6. Statistics

The average luminal cross-sectional area (volume/length) was correlated to the platelet (per unit graft length) end point data and fibrin end point data using SPSS (IBM, Version 24.0.0.0). *R*^2^ values were calculated for each material type (collagen ePTFE, ePTFE, and PVA), and an ANOVA was used to determine if the correlation was significant (*p* < 0.05). 

## 3. Results

### 3.1. Materials Processing: Thrombus and Lumen Identification to Generate Three-Dimensional Thrombus Models 

**Figure 4:**
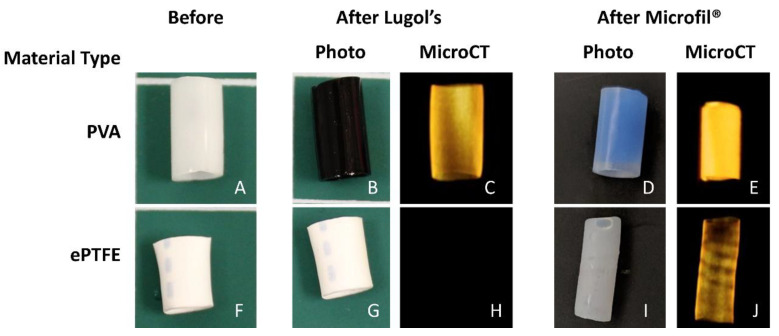
Results of material processing and microcomputed tomography (microCT) images for materials (poly(vinyl alcohol) (PVA) = (**A**–**E**), ePTFE = (**F**–**J**)) not exposed to blood. Samples were exposed to Lugol’s overnight (**B**,**C**,**G**,**H**), which was taken up by the PVA (**B**) and rendered the PVA radiopaque (**C**), but did not alter the ePTFE (**G**,**H**). Lumens of each material were filled with Microfil^®^ (**D**,**E**,**I**,**J**). Microfil^®^ stayed in the lumen of the PVA (**D**,**E**), but permeated the ePTFE (**I**), causing it to leak and making the walls of the material radiopaque (**J**).

The vascular graft samples without thrombosis testing were tested for radiopacity using multiple methods (Figure 4). The aqueous Lugol’s solution permeated the PVA hydrogels (Figure 4B,C) making the distinction of the thrombus and PVA graft material impossible during microCT imaging. Conversely, the Microfil^®^ solution remained within the lumen of the PVA graft and cured. For the ePTFE grafts, the ePTFE did not absorb the Lugol’s solution, (Figure 4H), making the open lumen and the graft itself distinguishable from the thrombus tissue during microCT imaging. When Microfil^®^ was used to fill the ePTFE grafts, there was significant leakage of the fill solution from the lumen of the graft (Figure 4J) due to ePTFE’s porosity. This resulted in the ePTFE material itself becoming radiopaque, and thus indistinguishable from the lumen during microCT imaging. On the basis of these results, Microfil^®^ was used to render the lumen of PVA samples radiopaque, and Lugol’s solution was used to render the thrombus of ePTFE and collagen ePTFE samples radiopaque.

**Figure 5:**
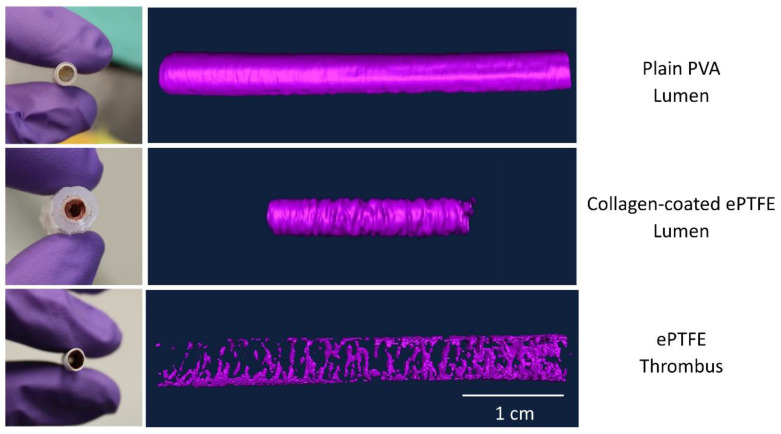
Generation of Amira models from microCT images. All grafts were 4–5 mm inner diameter. PVA and ePTFE samples were 3 or 4 cm in length. Collagen-coated ePTFE samples were 2 cm in length. Scale bar for model images = 1 cm.

In the presence of a thrombus, the material processing methods established above led to the greatest contrast for each material and thereby the most consistent quantification of the physical properties of the thrombus. Three-dimensional volumes of either the lumen (PVA, collagen coated ePTFE) or the thrombus (ePTFE) were generated for each graft type (Figure 5). 

### 3.2. Image Analysis: Validation 

The overall dataset presented here was completed by a single, blind observer. However, Amira image analysis and caliper measurement replicability were tested with three additional observers. Trained observers followed the guidelines in Figure 2, and were able to closely match the Amira volume data from the MicroCT images. Variability between users with the caliper measurements was tested with a data subset and generated a cross-sectional area range of 11.8–16.3 mm^2^ and a standard deviation range of 0.09–4.65.

**Figure 6:**
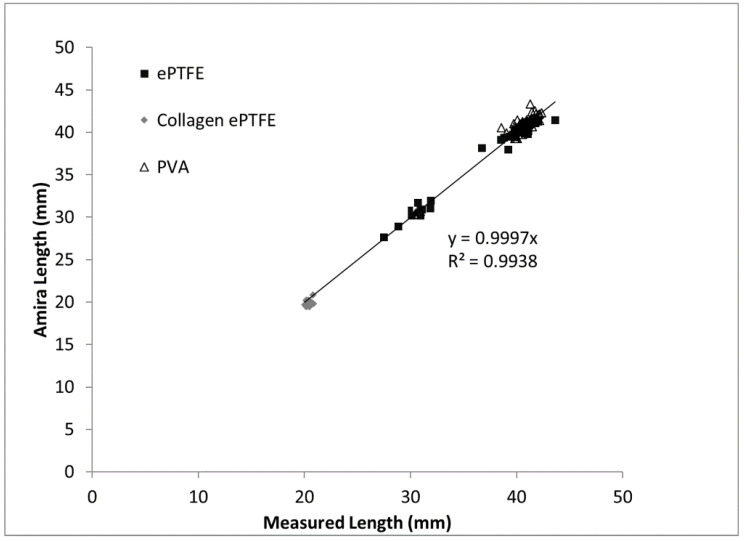
Comparison of caliper measured graft length to Amira-generated graft length for each material type.

The length of the final Amira surface was compared to the caliper measured length of the graft (Figure 6). Using the data depicted in Figure 6, a linear fit constrained to a *y*-intercept of (0,0) was generated. The linear fit had a slope of 0.9997, an *R*^2^ value of 0.994, and *p* < 0.0001. 

**Figure 7:**
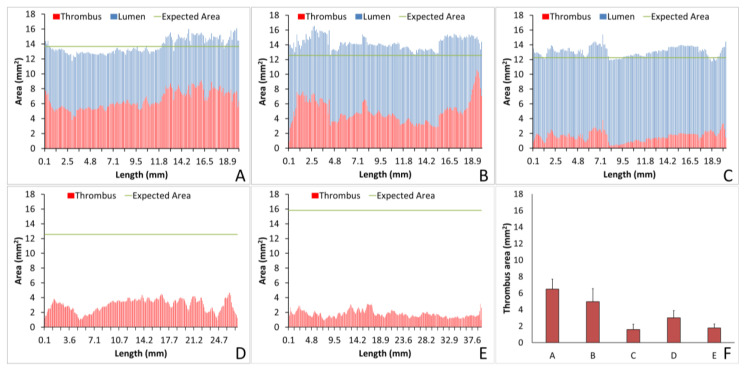
Quantification of the cross-sectional area per slice of the lumen or thrombus for single, representative samples, including a collagen-coated ePTFE sample without anti-coagulant (**A**), collagen-coated ePTFE with acetylsalicylic acid (ASA) alone (**B**), collagen-coated ePTFE with ASA and clopidigrel (**C**), ePTFE (**D**), and PVA (**E**). Horizontal lines in (**A**–**E**) labeled “Expected Area” are based on a caliper-measured diameter and represent a single point measurement. The average ± SD of thrombus area for all the slices is shown in samples (**A**–**E**) (**F**).

For the collagen-coated ePTFE samples, the luminal and thrombus areas were added together in each cross-sectional slice to validate both measurement types. Figure 7 depicts the Amira-measured thrombus area per cross-sectional imaging slice and the open luminal area per slice compared to the material’s calculated total cross-sectional area for a selection of representative samples. The material’s cross-sectional internal area was determined from the sample’s caliper-measured inner diameter. Collagen-coated ePTFE samples (Figure 7A–C), which were analyzed for both thrombus and luminal volumes, demonstrated the variability of a single thrombus and enabled confirmation of the method by comparing the lumen plus thrombus areas with the measured total area of a cross-section of a graft. The data in Figure 7A correspond to an overall thrombus volume of 6.49 ± 1.19 mm^2^ and a lumen volume of 7.24 ± 0.48 mm^2^. This analysis was also applied to the cross-sectional areas of each slice of the collagen-coated ePTFE grafts in animals pretreated with either ASA alone (Figure 7B; overall thrombus volume of 4.95 ± 1.59 mm^2^ and lumen volume of 9.32 ± 1.18 mm^2^) or dual anti-platelet therapy of ASA with clopidigrel (Figure 7C; overall thrombus volume of 1.59 ± 0.65 mm^2^ and lumen volume of 11.5 ± 0.50 mm^2^). The thrombus area of ePTFE samples (Figure 7D; overall thrombus volume of 3.01 ± 0.86 mm^2^) and PVA samples (Figure 7E; overall thrombus volume of 1.78 ± 0.48 mm^2^) were also represented on a per slice basis. This enabled analysis of the variability of individual thrombi (Figure 7F). 

### 3.3. Image Analysis: Thrombosis Comparisons

**Figure 8:**
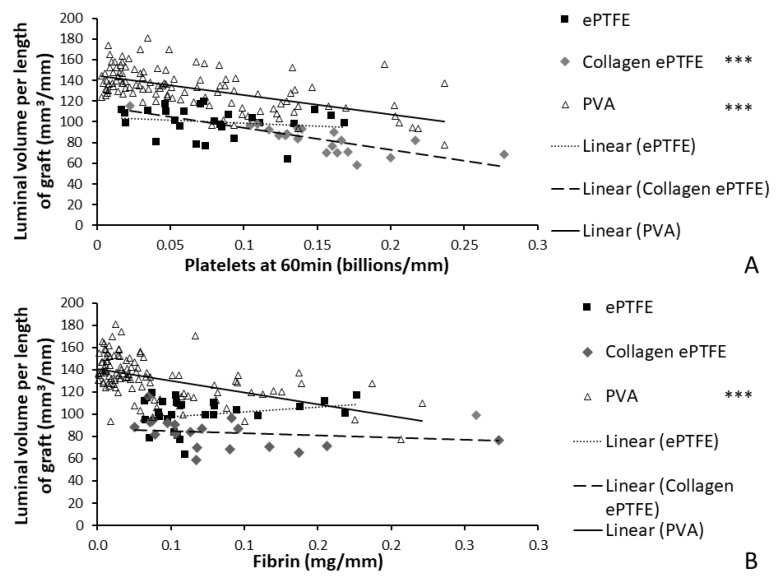
Validation of Amira quantification by correlation of luminal cross-sectional area with (**A**) platelet accumulation and (**B**) fibrin accumulation. *** indicates *p* < 0.001.

The platelet and fibrin accumulation data collected during the whole blood testing of 156 samples were compared to the average luminal cross-section measured using the Amira software for each material analysis type. The use of multiple materials and anti-coagulant test conditions produced a spread of platelet accumulation between 0.0030 and 0.28 billion/mm length of graft. For the PVA with prothrombotic surface coatings and the collagen-coated ePTFE, where substantial platelet accumulation occurred, there were significant inverse correlations between the platelet count and the open luminal volumes for the grafts. Platelet accumulation (Figure 8A) was highly significant for PVA samples (*R*^2^ = 0.341, *p* < 0.001, and *F* = 56.370) and collagen-coated ePTFE samples (*R*^2^ = 0.618, *p* < 0.001, and *F* = 29.175). The ePTFE subset did not show significant correlation between platelet accumulation and average cross-sectional luminal area (*R*^2^ = 0.032, *p* = 0.381, and *F* = 0.796).

For the PVA grafts, there was a strong inverse correlation between the fibrin accumulation data (Figure 8B) and luminal area in that material type (*R*^2^ = 0.245, *p* < 0.001, *F* = 35.328). Unlike in the comparison to platelet accumulation, the regression of average cross-sectional luminal area of collagen-coated ePTFE samples with fibrin accumulation was not significant (*R*^2^ = 0.039, *p* = 0.402, *F* = 0.737). The ePTFE subset regression was also not statistically significant for fibrin accumulation (*R*^2^ = 0.070, *p* = 0.191, *F* = 1.812). 

### 3.4. Implant Testing

**Figure 9:**
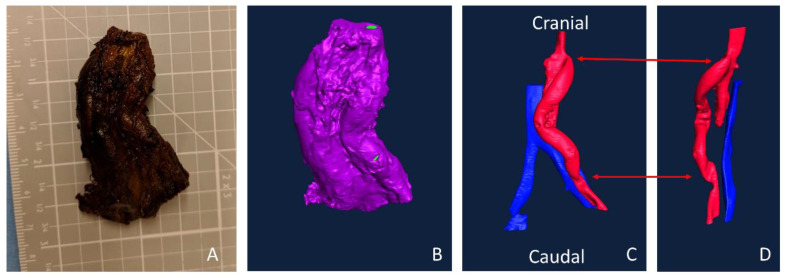
Explanted aorto-iliac bypass sample after 28-day implant. The ePTFE sample was implanted on the right side, and after Lugol’s soaking (**A**), the surrounding tissue was rendered radiopaque. After microCT imaging and Amira surface rendering (**B**), open lumens were selected. The aorta and graft, in red, show the branching from the abdominal aorta to the iliac artery (**C,D**). The aorta is tied off and stops in (**D**) but would follow a similar anatomical path—parallel to the vein (blue), which was unaltered during surgery. Arrows indicate the position of the anastomoses: proximal at the top/cranial, distal at the bottom/caudal.

Lugol’s soaking rendered all the tissue radiopaque for microCT imaging (Figure 9A,B), allowing the open lumens to be clearly distinguished during image analysis (Figure 9C,D). Only the ePTFE graft (Figure 9, right side) remained patent during this implant due to surgical complications; the lumen of this graft was clearly visible, connecting the aorta to the iliac artery. The graft illustrated a tortuous path, which could be viewed without dissection of the surrounding tissue (and potential loss of that structural integrity). Although the exact position of each of the anastomoses is difficult to quantify, it appears that some narrowing occurred there.

## 4. Discussion

This work presents a broadly applicable technique for quantifying the physical characteristics of biomaterial-induced thrombosis, which can translate to an improved understanding of clinical patency for cardiovascular device development. Thrombosis research is typically limited to specific clotting characteristics (e.g., platelet attachment, FXa (activated factor X) generation), but even when fresh, flowing, whole blood is used, little emphasis has been given to the physical characteristics of the formed thrombus beyond basic observation. These thrombus characteristics can dictate the percent stenosis and ultimately the success or failure of the device. This approach addresses the limited understanding of physical thrombus characteristics in vascular grafts with a variety of biomaterials.

The goal of this study was to improve upon existing methods for assessing the thrombogenicity of cardiovascular materials by developing and validating an imaging-based method that provides detailed information about the physical parameters and gross morphology of thrombi formed under flowing whole blood. The thrombus volume data obtained by applying the microCT and image analysis method to a large combination of biomaterials, surface modifications, and systemic therapeutics indicate the technique’s broad applicability. The correlation of the measured thrombus volumes to the existing platelet and fibrin amounts supports the accuracy of the method. Among the validating metrics presented in this study are the comparison of the Amira-measured graft lengths to the caliper-measured lengths. As would be expected, the length measurements exhibited a near-perfect one-to-one correspondence, with a strong statistical correlation and a trendline slope of almost exactly one. Therefore, on the basis of the results of the study, a novel methodology has been developed to provide key quantitative metrics of the formation of thrombi on blood-contacting biomaterials under flow.

The developed method included distinct material processing techniques for different biomaterials, selected on the basis of their material properties. The overall goal was to generate differences in radiopacity between the lumen, the thrombus, and the biomaterial. This contrast between lumen and thrombus in the microCT images was necessary for the subsequent detection and quantification by the image analysis software. In this study, hydrogels (PVA) and non-woven meshes (ePTFE) were tested. However, these techniques can be translated to a vast array of biomaterials. For example, the technique of soaking materials in aqueous Lugol’s solution can be extended to other hydrophobic materials. Although the hydrophilicity of PVA caused equivalent uptake of Lugol’s solution by both the thrombus and the graft biomaterial, perfusing the lumen with Microfil^®^ enabled a clear distinction of the lumen. Therefore, this use of Microfil^®^ provides a material processing technique that can be translated to other hydrogels and nonporous materials [24].

The materials tested during the development of this method span the spectrum of thrombogenicity, from low platelet attachment (as low as 0.003 billion platelets per mm) on some PVA grafts, up to 0.28 billion platelets per millimeter of graft length on collagen-coated ePTFE after 60 min exposure to whole blood. This large range of responses was designed into the study by using multiple materials, testing with and without antiplatelet therapies, and using multiple surface modifications of the PVA. The various PVA modifications were not illustrated in the current work to compare their effects, but rather were merged into a single dataset to generate a broad response range. Overall, this broad response suggests the usefulness of this technique to quantify the physical parameters of the thrombus, which can be applied to materials with a varying degree of hemocompatibility. Because platelets and fibrin are key components of thrombi formed under flow, the measured elevated amounts of platelets and fibrin on the graft surfaces during the whole blood testing indicate the formation of a thrombus, and a resultant reduction in luminal area. Therefore, the negative slope of the correlations between luminal area and platelet accumulation on the PVA and collagen-coated ePTFE grafts provides a particularly strong validation of this technique (Figure 8). It is notable, however, that the comparison of the luminal area of collagen-coated ePTFE grafts with fibrin accumulation did not demonstrate a significant correlation (Figure 8; *R*^2^ = 0.039, *p* = 0.402, *F* = 0.737). This may have been due to the orientation of the hydroxyl groups in the fibrinogen protein, which render it more likely to interact with PVA surfaces than collagen-coated ePTFE surfaces [24,25]. Importantly, these whole blood experiments occurred under an arterial flow rate, which tends to lead to the formation of more platelet-rich thrombi. In contrast, thrombi formed under venous flow tend to contain higher fibrin quantities [26,27,28]. The application and correlation of this method of thrombus quantification to platelet and fibrin accumulation remains to be performed under venous flow conditions.

The application of an imaging-based method to the measurement of thrombus formation on relatively non-thrombogenic cardiovascular biomaterials presents broader challenges. This was demonstrated in this study’s finding of a relatively weak correlation between the resultant luminal area and the platelet and fibrin attachment measured on ePTFE grafts (Figure 8). The PVA (with a broad range of responses due to the numerous surface modifications) and collagen-coated ePTFE (inherently prothrombogenic) biomaterials had strong correlations with an increase in platelet and fibrin accumulation, indicating a decrease in average luminal area. In contrast, the average luminal area in ePTFE samples did not change dramatically across a broad range of platelet and fibrin attachment, leading to the lack of correlation. Although this weak correlation could be attributed to a greater platelet and fibrin composition in the ePTFE thrombus without as much of an effect on the luminal area, the relative inefficacy of the method as applied to non-collagen coated ePTFE grafts is hypothesized to be caused by the limited ability of the user to distinguish small differences in pixel intensity when a low quantity of pixels is present, as in the ePTFE with low thrombogenicity. In order to determine if the unchanged luminal area is due to thrombus composition or a limitation in resolving small thrombus areas, future experiments could adopt a longer whole blood testing study period. Additionally, different microCT instrumentation with higher resolution would enable the quantification of smaller thrombi or thrombi in smaller diameter grafts. Indeed, we have been successful at translating these protocols and image analysis method to an alternative microCT system, and anticipate that researchers could use any microCT imaging system that this available to them. Additionally, alternative contrast agents, such as nanoparticles [29], may improve feature detection and improve image segmentation. 

The analysis of individual thrombi on grafts can provide valuable information on the hemocompatibility of biomaterials. Using Amira, we were able to quantify the lumen or thrombus cross-sectional area per slice, enabling analysis of the thrombus formation at all points throughout the graft and a quantification of the thrombus variability across the graft length. For example, for the collagen-coated ePTFE grafts tested under no anticoagulant and ASA alone, the average thrombus areas were equivalent, as shown in Figure 7A,B, respectively. Importantly, however, the ASA treated study resulted in an increased variability of cross-sectional thrombus volume per slice. Physiologically, this could predispose the thrombus to embolization. Although the data presented here only display a representative image and a full analysis is beyond the scope of presenting this method, future work will examine the variability and the compare distributions between various samples. In addition to improving the understanding of biomaterial hemocompatibility in the well-established and well-controlled arteriovenous, whole blood, shunt model, these methods illustrate the potential of applying this method to a more complex in vivo, end-to-side implant model. Although the occluded sample could not be visualized with this technique, the patent side was well illustrated. Using non-destructive imaging at this length scale allows for an increased understanding of the path of the blood flow and the patency of the implanted graft along its length and particularly at the critical points of healing, the anastomoses. The sample tested here did suggest some narrowing at the anastomoses. This imaging could be combined with the more destructive histological process to quantify the intimal hyperplasia at the anastomoses [8]. Thus, these methods could be used to characterize both the hemocompatibility of biomaterials, as well as the healing responses, therefore identifying where biomaterials fail.

The methods developed in this study do have some limitations. The specific equipment (microCT scanner and Amira software) require a significant financial investment, and the training of Amira program users to accurately determine pixel masking ranges is time-intensive. However, the methods developed and presented in this study provide novel, validated techniques for the measurement of the physical parameters of thrombi formed on blood-contacting materials under flow. Applications of this technique to future work could include its extension to the hemocompatibility of biomaterials under venous blood flow conditions, and to the development of novel cardiovascular biomaterials and tissue-engineered implants. Given the well-established importance of assessing biomaterial thrombogenicity prior to clinical implementation, the introduction of this methodology provides a powerful tool for the development of blood-contacting biomaterials and tissue-engineered implants.

## Figures and Tables

**Table 1:** Summary of material processing. Biomaterials were rendered radiopaque in different ways on the basis of their material properties.

Material Type	Graft Processing	Amira Selection(s)
PVA	Microfil^®^	Lumen
Collagen ePTFE (positive control)	Lugol’s	Lumen and thrombus
ePTFE (clinical control)	Lugol’s	Thrombus
